# Smart-phone, paper-based fluorescent sensor for ultra-low inorganic phosphate detection in environmental samples

**DOI:** 10.1038/s41378-019-0096-8

**Published:** 2019-10-21

**Authors:** Mehenur Sarwar, Jared Leichner, Ghinwa M. Naja, Chen-Zhong Li

**Affiliations:** 1 Nanobioengineering/Bioelectronics Laboratory, Department of Biomedical Engineering, Florida International University, 10555 West Flagler Street, Miami, FL 33174 USA; 2 Everglades Foundation, 18001 Old Culter Road, Palmetto Bay, FL 33157 USA

**Keywords:** Other photonics, Optics and photonics

## Abstract

A major goal of environmental agencies today is to conduct point-of-collection monitoring of excess inorganic phosphate (Pi) in environmental water samples for tracking aquatic “dead zones” caused by algae blooms. However, there are no existing commercial devices which have been miniaturized and are suitable for the point-of-need-testing (“PONT”) that is required to fully map a large region, such as the Florida Everglades. To solve this challenge, a reflection-mode fluorescence-sensing apparatus was developed, leveraging an environmentally sensitive fluorophore (MDCC) bound to a bacterial phosphate-binding protein to generate a fluorescent optical signal proportional to the concentration of (Pi) present. The combined end-to-end integrated sensor system had a response time of only 4 s, with minimal effects of common interfering agents and a linear range spanning from 1.1 to 64 ppb. To support ease-of-use during PONT, the platform incorporated disposable wax-printed paper strip sample pads and a smartphone camera detection system. Since the EPA threshold is currently 30 ppb to prevent eutrophication, this system serves as a rapid test of whether a region is compliant.

## A simple sensor for finding phosphates

A disposable phosphate sensor that can be imaged directly with a smartphone could offer a simple and effective strategy for monitoring water contamination. The phosphates present in agricultural fertilizer can create an environmental disaster if they spill into surrounding water, producing algal ‘dead zones’. Standard methods for measuring inorganic phosphate are time-consuming and employ toxic chemicals, but researchers led by Chen-zhong Li at Florida International University (FIU) have developed a potential alternative for onsite testing. Teaming up with the Everglades Foundation, Li’s lab developed a paper-based sensor incorporating a fluorescently labeled bacterial protein, which generates a rapid visual readout upon binding to inorganic phosphate. Their sensor is capable of detecting whether phosphate levels in a water sample exceed legal limits and can be analyzed with a standard smartphone, making it a promising strategy for assessing contamination in the field.

## Graphical abstract

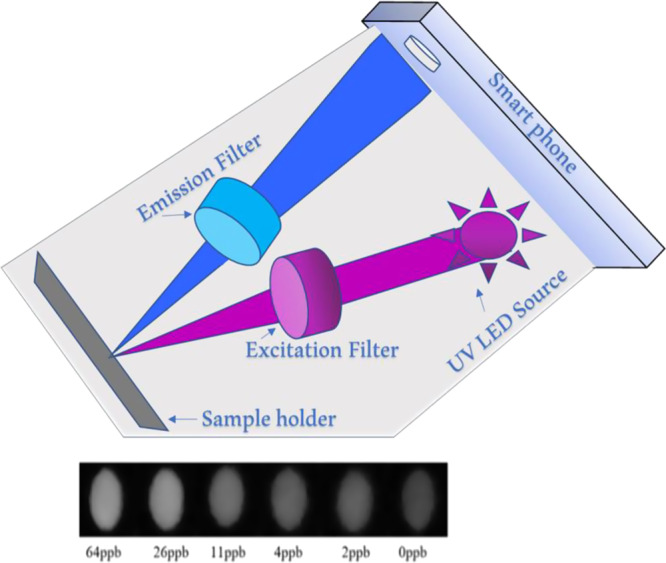

## Introduction

High levels of inorganic phosphate (Pi) are one of the main causes of the growth and spread of environmentally detrimental algae^1^. Phosphate is a nutrient which acts as a growth limiting factor for living organisms. In the presence of warm water, phosphate can multiply and spread toxic algae uncontrollably, enveloping the surface of any water body. According to a report by the National Oceanic and Atmospheric Administration (NOAA), up to nine inches of heavy blue–green algae bloom has been found in Lake Okeechobee in Florida after heavy rain^2–4^. Scientists are expecting more blooms as atmospheric temperatures rise due to global warming^5–7^. Despite these deleterious effects, phosphorous is a crucial element for the growth of plants and animals, as well as in lake ecosystems^8^. Plants and animals both require it due to their dependence on the Krebs cycle for the production of energy, as it produces guanosine triphosphate (GTP) by a cascade of chemical reactions. Therefore, water which is deficient in phosphorus is similarly detrimental for a healthy freshwater system.

In nature, phosphate exists in either an inorganic or an organic state^9^. Within the inorganic phosphates, further subdivision exists based on the number of phosphate atoms in the molecule, with orthophosphates representing a single phosphorus atom, pyrophosphates representing two phosphorus atoms, and polyphosphates representing all molecules with additional phosphorus atoms in their structure. Based on the pH level of the water, orthophosphates exist as a distribution of H_3_PO_4_, H_2_PO_4_^−^, HPO_4_^2−^, and PO_4_^3−^. Orthophosphate levels are strongly affected by local agricultural runoff. Organic phosphates present in these bodies of water also include phospholipids and nucleic acids (e.g., from plant tissue) and organophosphorus pesticides, but these are not detected by the proposed system. Inorganic phosphates can be easily utilized by the living organisms, thus is the major contributor to dangerous algae blooms. Hence, the developed device can serve an important function for monitoring or predicting these events.

Within Florida, 400,000 acres land is being used for growing sugarcane, and phosphorus is a predominant component of the fertilizer used in caring for the crops. Recommendation for applying phosphorus range from 0 to 75 pounds P_2_O_5_ per acre^10^. In an assessment run by the University of Florida, the total phosphate found in sugarcane cultivated soil was 1227 parts-per-million (ppm), whereas it was 959 ppm in uncultivated soil. Heavy rainwater erodes this soil and flushes this phosphate-rich liquid into the downstream water supply. In terrestrial settings, (Pi) concentrations exist in the higher ppm range, but due to this runoff into bodies of water, the concentration naturally diminishes into the parts-per-billion (ppb) range due to the large volume of water it is being dissolved into. This ppb range is the one currently of interest to various environmental monitoring groups, since it is the most appropriate scale for assessing aqueous (Pi) contamination and is ideal for predicting algae blooms. Sensors capable of ppm measurements, which are much more common, are not sensitive enough to detect these aqueous levels of inorganic phosphate. Thus, it is important for environmental chemists to be able to monitor local (Pi) levels at these low concentrations in order to identify “hotspots” and handle them accordingly. Currently, the EPA has decided that (Pi) cannot exceed 30 ppb in environmental bodies of water^11^.

While the Florida Everglades is a prime target for (Pi) monitoring efforts, the impacts of phosphorus pollution in water are far more widespread. Current estimates of impacts of (Pi) contamination on the U.S. economy describe it causing losses of $2 billion per year^12^. This substantial cost is driven by expensive water treatments, falling population of aquatic species, and devaluation of properties located close to contaminated areas. With growing global temperatures, the issue of algae blooms is not only limited to the United States. Immediately prior to the 2008 Olympics, a widespread algae bloom in Qingdao, China required an estimated $592,657 million CNY for resulting remediation operations^13^. Furthermore, widespread eutrophication of Switzerland’s Lake Constance cost $2.6 billion and ~40 years to return it to a healthy condition^14^. Clearly, the impacts of (Pi) pollution are felt widespread.

A historical method for the laboratory grade determination of (Pi) uses ammonium molybdate, which reacts with (Pi) to form a blue-colored phosphomolybdate complex. The absorbance of the complex is proportional to the level of inorganic phosphate^15–17^. The limitations of this approach include reading error from interfering agents like arsenates, silicates, sulfides, and oxidizing agents^18^. Few commercial products also use this methodology, however, since it requires a continuous supply of reagents and cuvettes. In addition, reagents are extremely toxic and should not be handled at the point-of-testing to prevent contamination of chemicals into the water stream and the response time is very slow^19^. Thus, the process does not allow for a convenient testing process while in the environmental setting. Unfortunately, despite these issues, this historical approach, first developed in the 1980s, is the only methodology the EPA currently utilizes^20^.

A wide variety of techniques have been used in modern (Pi) sensors, spanning both electrochemical and optical modalities. Within electrochemical techniques, potentiometric strategies measure the electrical potential between two electrodes and utilize chemical reactivity of an analyte as a tool to influence the electrical potential. Modern potentiometric sensors often use Cobalt as an electrode material since the precipitation of phosphorus on its surface shifts the potential to be more electronegative^21^. The concept has been further miniaturized through the incorporation with screen printing^22^, but all Cobalt-based sensors suffer the fundamental limitation of interference by dissolved oxygen and chloride ions, limiting their usability in aquatic environments. Amperometric sensors measure current flow under a fixed electrical potential, taking advantage of oxidation and reduction reactions involving the analyte of interest to modify current flow proportionally to concentration. Modern amperometric techniques similarly utilize Cobalt^23^ due to its reactivity with inorganic phosphate. Voltammetric techniques measure current flow under varying potential, providing measurement at far lower concentrations at a much higher technological cost. Screen-printed^24^ and Molybdenum-based^25^ methodologies have substantially reduced the detection limit from the previous techniques. Conductance is a simpler and lower-cost measurement strategy, but at the cost of sensitivity^26^. In contrast, capacitance techniques are capable of substantially high sensitivity at a low cost, but are highly sensitive to varying environmental factors—making them unsuitable for field measurements^27^.

In contrast to these modern electrochemical strategies, optical techniques for (Pi) measurement are largely limited to colorimetric/absorbance or fluorescent techniques (Table 1). Modern colorimetric/absorbance sensors are typically only improvements over the phosphomolybdate assay^28^, and are thus also subject to its many chemical interferences. Fluorescence approaches which have been recently leveraged include utilizing the inorganic phosphate-sensing ability of the flavonoid morin in conjunction with the fluorescence capabilities of an aluminum–morin complex^29^. While this approach had only minor interference, it was not sensitive enough to provide the necessary low-ppb measurements. Alternative fluorescence techniques include the utilization of the fluorescence-quenching effect of Europium when bound to fluorescent graphene quantum dots^30^. The presence of (Pi) solution detaches this complex, restoring fluorescence proportion to the (Pi) concentration. This technique similarly had very low interference and further improved upon the sensitivity as compared with the previous aluminum–morin complex. The low interference of fluorescent technique was the primary justification in pursuing a fluorescent strategy, and as the above table documents, the current work was able to further reduce the measurement range to the necessary sensitivity to encompass existing EPA pollution thresholds.

**Table 1:** Comparison of modern inorganic phosphate sensing technologies

Detection technique	Sensor description	Range (ppm)	LOD (ppm)	Interferences	Response time	Reference
Electrochemical (*Potentiometric*)	Cobalt-based screen-printed electrode	39–3096	31	Dissolved oxygen	40 s	^22^
Electrochemical (*Amperometric*)	Cobalt–copper Electrode	39–3096	Not reported	Oxygen interference reduced through use of copper	<30 s	^23^
Electrochemical (voltammetric)	Paper-based screen-printed electrode	0.31–9.3	0.12	Not reported	Not reported	^24^
Electrochemical (voltammetric)	Metallic molybdenum electrodes	Mode 1: 0.0031–0.031 Mode 2: 0.008–0.12	Mode 1: 0.0015 Mode 2:0.0031	No silicate interference	Mode 1: 60 mins Mode 2: 30 mins	^25^
Electrochemical (*Conductance*)	Molecularly imprinted polymer	0.66–8	0.16	Alkalinity and anions can Interfere	2 min	^26^
Electrochemical (*Capacitance*)	Phthalocyanine-acrylate polymer adduct	0.000003–0.3	0.00003	Low interference by chloride, sulfates, and carbonates	Not reported	^27^
Optical (*Colorimetric*)	Molybdenum phosphate-blue assay	0.004–0.31	0.001	Significant arsenic and silicate interference	5 min	^28^
Optical (*Fluorescence*)	Aluminum–morin microspheres	0.1–1	0.1	Low interference by nitrate, carbonate, and sulfate	5 min	^29^
Optical (*Fluorescence*)	Graphene quantum dots	0.031–0.4	0.003	Low interference by nitrates, F^−^, and chloride	Not reported	^30^
Optical (*Fluorescence*)	Disposable wax-printed paper strip sample pad in 3D-printed smartphone sensing apparatus	0.001–0.064	0.001	Low interference from NaCl (0.02%), F^−^ (4.09%), MgCl_2_ (3.78%), NO_3_ (3.21%), and KCl (3.93%)	4 s	Proposed work

**Fig. 1:**
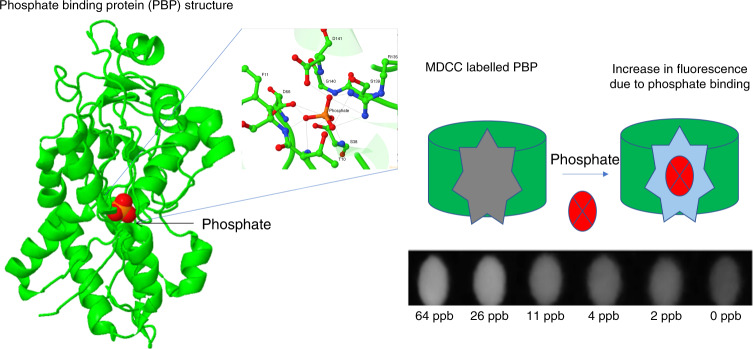
(Top) Illustration of (Pi) interaction with MDCC bound phosphate-binding protein. (Bottom) Representative images of our experimental data demonstrating the variation of fluorescent intensity with (Pi) concentration

In order to leverage the advantages of fluorescent techniques, we utilized the substantial specificity of *E. coli* phosphate-binding protein (PBP). The interaction between the phosphate-binding protein and (Pi) is rapid, with an extremely high affinity (*K*_d_ = 0.1 µM). This PBP was bound to MDCC, a thiol-reactive coumarin, whose more detailed name is N-[2-(1-maleimidyl)ethyl]−7-(diethylamino)-coumarin-3-carboxamide (MDCC)^31^. The coumarin group has the capability of monitoring conformational changes to the molecule, which subsequently affects the intensity of fluorescence emission. Upon binding of (Pi) to the cleft of the protein, the bound MDCC fluorophore shows increased emission of fluorescence at 455 nm when excited at 410–420 nm. This nature of the protein can be exploited to develop a strong chemical sensor to specifically identify (Pi) in the water sample. The interaction of the MDCC-bound protein with (Pi) is depicted in Fig. 1 below, demonstrating both the binding cleft and resulting concentration-dependent fluorescence response.

This PBP–MDCC complex has been established in the past and utilized for cellular-scale (Pi) measurements, but until now was unable to be adapted for a portable assay for environmental monitoring. In order to minimize the problems of batch-to-batch variability of synthesis while ensuring that other researchers could rapidly reproduce our methods without needing to invest in the considerable equipment for bacterial culture, we chose to incorporate a recently commercialized version of this conjugate. This product, released by ThermoFisher Scientific, is composed of a purified recombinant *E. coli* PBP with a labeled MDCC at the A197C position. The conjugated pair has an extinction coefficient of 68,350 M^−1^ cm^−1^ with an overall dye to protein ratio of over 96%. The sensor was stored in 10 mM Tris–HCl (pH 7.6) buffer with 50 mM NaCl until use.

(Pi) contamination is a serious and growing problem, only further exacerbated by the growing human population. The subsequent impacts of eutrophication are costly, create health risks, and are present around the world. Many historic and modern techniques for measurement are available, but fluorescence-based measurement has emerged as a powerful technique due to its enhanced specificity and sensitivity and less interference potential when compared with common electrochemical strategies. Our proposed device not only further improves upon existing sensitivity but also is encapsulated into a convenient portable package with disposable paper strip sample pads for field use. Characterization and optimization of the device encompassed the development of a five-point assay for field use, assessment of photobleaching, determination of interfering agents, and testing against actual environmental samples.

## Results

To characterize the composite device, a variety of tests were conducted. First, a five-point assay was constructed to allow for the generation of a simple linear curve within the range of the device’s capabilities during field measurements. In addition, a photobleaching assay was conducted in order to identify the impact of excitation light exposure on measurement outcomes. Furthermore, potential interfering agents were tested against device performance, at concentrations above the highest expected concentrations. Finally, device performance was tested against field samples in conjunction with measurement by government agencies, as well as a modernized commercial device.

**Fig. 2:**
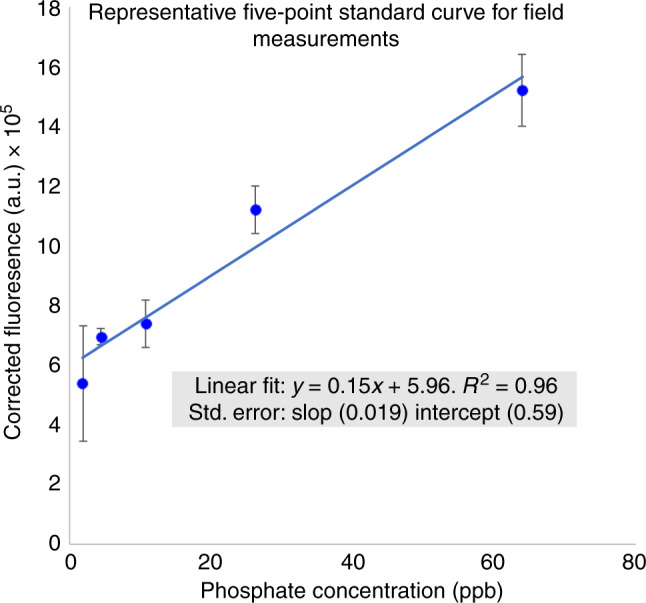
Representative five-point standard curve from a handheld smartphone device for field measurements. Error bars represent standard error. Corrected fluorescence intensity is a metric which subtracts the average intensity in the desired measurement zone by the average intensity in a background zone^32^. This technique minimizes measurement error due to fluctuations in the light source intensity or camera performance

Development of a five-point standard curve was optimized to provide sufficient sample size for accurate linear cure development while providing enhanced sample density at lower concentrations. As shown below in Fig. 2, a typical standard curve used to calibrate device functionality would provide a high correlation (*R*^2^ = 0.96) between 2 and 64 ppb. The pixel values were transformed into the units of corrected fluorescence intensity^32^. The limit of detection, 1.1, was determined through a serial dilution method which tested whether the fluorescence of the current and previous sample differed with statistical significance.

The liquid sensor was also validated by use within a Bio-Tek microplate reader, and was found to have a lower limit of detection, 150 ppt, and a broader linear range, up to 80 ppb. However, it is important to recognize that this microplate reader costs tens of thousands of dollars, while the total cost of the handheld device is merely a couple of hundred dollars without the smartphone.

**Fig. 3:**
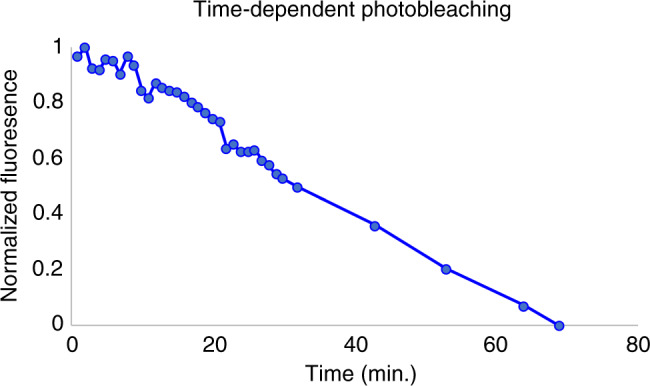
Photobleaching test, demonstrating loss of fluorescent signal as a function of continuous illumination. Inset figure provides a zoomed perspective of the first 10 min. Since only 4 s of illumination is required per measurement and each strip can only be used once, we do not believe photobleaching will substantially affect our outcomes

Another important test that was carried out was an assessment of the photobleaching effect of the fluorescent protein. This was essential, as even though the reading can be taken after only 4 s of illumination, the high-power LED has the potential to photobleach the fluorescent probe rapidly. Due to the high-power nature of the utilized LED coupled with the high-intensity focusing point, it became important to assess whether photobleaching played a role during experiments. To identify whether this was an issue, a fluorescently prepared sample with a high concentration of Pi was placed under constant illumination, with numerous images taken at various time points over a 70-min period. During typical measurements, samples are only illuminated for 4 s, and are not reused. A significant change in intensity was only found after 5 min, as shown below in Fig. 3, which is far less than the illumination that would be required during a single trial.

**Fig. 4:**
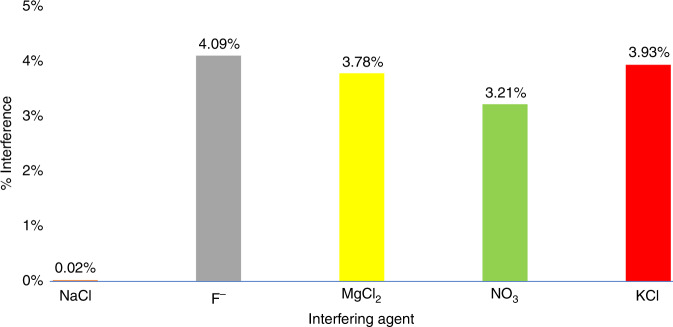
Observed interference from known interfering agents

Finally, the interference of common ions present in the Everglades water^33^ samples were assessed to identify suitable targets for an interfering agents study. To perform this experiment, the fluorescence was measured at different concentrations of inorganic phosphate, and for each condition, a trial was performed with and without the interfering agent. The percentage difference between the collected fluorescence values provides an indication as to the interference of the ion within the assay. The addition of each agent reduced the fluorescence slightly when compared with (Pi) alone, as demonstrated below in Fig. 4. NaCl reduces the signal 0.02%, which is much lower than that of F^−^ (4.09%), MgCl_2_ (3.78%), NO_3_ (3.21%), and KCl (3.93%). It is fortunate that NaCl has such a minimal effect since the Everglades has both freshwater, saltwater, and brackish water^34^. In addition, the other ions tested, which are common interfering components for (Pi) assays, were shown to only generate minimal interference. Further details can be found in the Materials and methods section.

The device was also tested against actual samples from the Everglades from two different geographic locations. In each instance, the concentration reported by the Everglades Foundation using their established assay generated “negative values”, since it went below their internal linear curve. This indicates the true need for this device within the field, as established methods used by environmental organizations are simply unable to accurately measure such low concentrations. The developed device, however, was able to generate a measurement within its linear range. Samples were collected from two locations (“NP201” and “P36”) in the Everglades via helicopter, and a Liter of surface water was collected. The sample was thoroughly vortexed to maximize heterogeneity and an 18-µl sample was dispensed onto the paper strip substrate, which already contained 2 µl of the fluorescent probe, physically adsorbed onto the individual cellulose fibers. A linear curve was first generated using six unique concentrations of phosphate standard, with three repetitions of each condition. Afterward, multiple samples of each environmental sample were prepared and imaged. In order to determine the accuracy of our measurement, a commercialized device, the Hanna Phosphorus Sensor, was also used to measure the environmental samples. This commercialized device is not suitable for field use due to its long response time, the requirement of excessive glassware, toxic powder substrate, and intrinsic measurement variability.

**Fig. 5:**
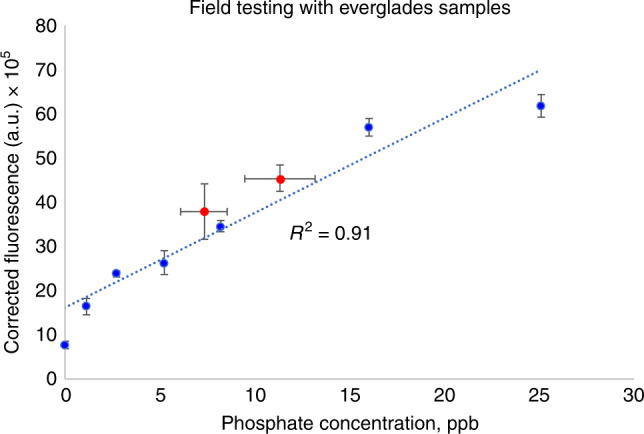
To perform field testing experiments, a linear curve is first constructed with standard solutions (blue dots, dotted blue line). The standard solution concentration is plotted as the *x*-axis value and the correct fluorescence is plotted along the *y*-axis. Following this, experimental samples from the Everglades were tested with our system (red dots). To validate the results, a commercial measurement system was used in parallel. These experimental samples from the P36 and NP201 regions are plotted in red, with the *x*-axis representing measurement by the commercial device and the *y*-axis representing the corrected fluorescence from our optical system. From these experimental samples, horizontal error bars represent the standard error of the commercial (Pi) sensing device, while vertical error bars represent the standard error of our fluorescent measurement scheme. The close proximity of these points to the established linear curve and the high overall correlation of the linear fit combining standard and experimental samples demonstrates the suitable performance of the system

In Fig. 5 below, we documented the generated linear curve (blue points) alongside the measured environmental samples (red points). For the environmental samples, values were plotted based on the corrected fluorescence value (*y*-axis) and the measured (Pi) concentration from the Hanna commercialized device (*x*-axis). Further details are provided in the caption. The close proximity of the environmental sample points to the linear curve demonstrates similar measurements from both devices.

## Discussion

Based on these findings, it is readily apparent that this device holds the potential to provide a solution for environmental biologists seeking to track (Pi) contamination in the aquatic environment. Not only does it provide near-instantaneous results and work with a low-cost disposable paper strip platform, but results can also be rapidly geo-tagged for later GIS mapping of (Pi) distribution. Current methods of detecting Pi typically suffer from a combination of three problems—requiring expensive, bulky laboratory equipment within a controlled setting, not being able to detect (Pi) in low enough levels and having long response times. This proposed solution aims to eliminate these challenges and pave the way for improved Pi sensing that can finally rapidly answer a key question—whether a collected sample falls below or above the EPA threshold, 30 ppb. Previous studies examining the affinity and fluorescence changes of the *E. coli* PBP caused by variations in pH have identified a reduction in the fluorescence intensity as the pH increases above 8^35^. However, we are not concerned about this significantly impacting our results since no significant pH-dependent differences were found between 6.5, 7, and 7.5, and only identified substantial impacts when the pH rose to 8 or above. When assessing pH stability of Everglades surface water, recent studies have found it to remain in a tight range between 7.0 and 7.2^36^, which was chosen for our analysis. It will be extremely important to monitor this parameter during field testing.

A fundamental limitation of this work is the difficulty in ensuring that any residual (Pi) contamination remained far below the relevant analyte concentrations being measured. Unfortunately, (Pi) is often present, adsorbed onto the surface of all plasticware, glassware, and transfer tools. Thus, a rigorous washing routine was carried out utilizing ultrapure DI water at every stage of sample preparation. Due to the omnipotent presence of trace inorganic phosphate, the proposed assay will always suffer from variability, however, small, resulting from the widespread (Pi) contamination in these small quantities.

It is important to recognize that unlike electrical approaches, our optical approach utilizing the highly specific PBP provides us far less likelihood of ionic interference than a comparable electrical sensing method. Examining the current governmental standard methods, the Ammonium Molybdate method is interfered with by silica and arsenate^37^, while the Malachite Green method is interfered with significantly by carboxylic acid salts and detergents^38^. The PBP used in the reported design does not form an intermediate susceptible to interference and instead only interacts significantly with inorganic phosphate. Despite the additional quantification complexity of fluorescence techniques, we believe this composite device is a highly suitable target for field measurements.

## Materials and methods

The underlying methodology of this study can be simplified to a few core components. First, the measurement apparatus was designed to hold all required optical components and 3D-printed. Second, the wax-printed chromatographic sample pad was designed and printed. Third, the fluorescent complex was adsorbed onto the surface of the sample pad. Finally, the calibration of smartphone imaging parameters was undertaken to maximize imaging sensitivity. The strategies for each of these steps will be discussed in turn.

**Fig. 6:**
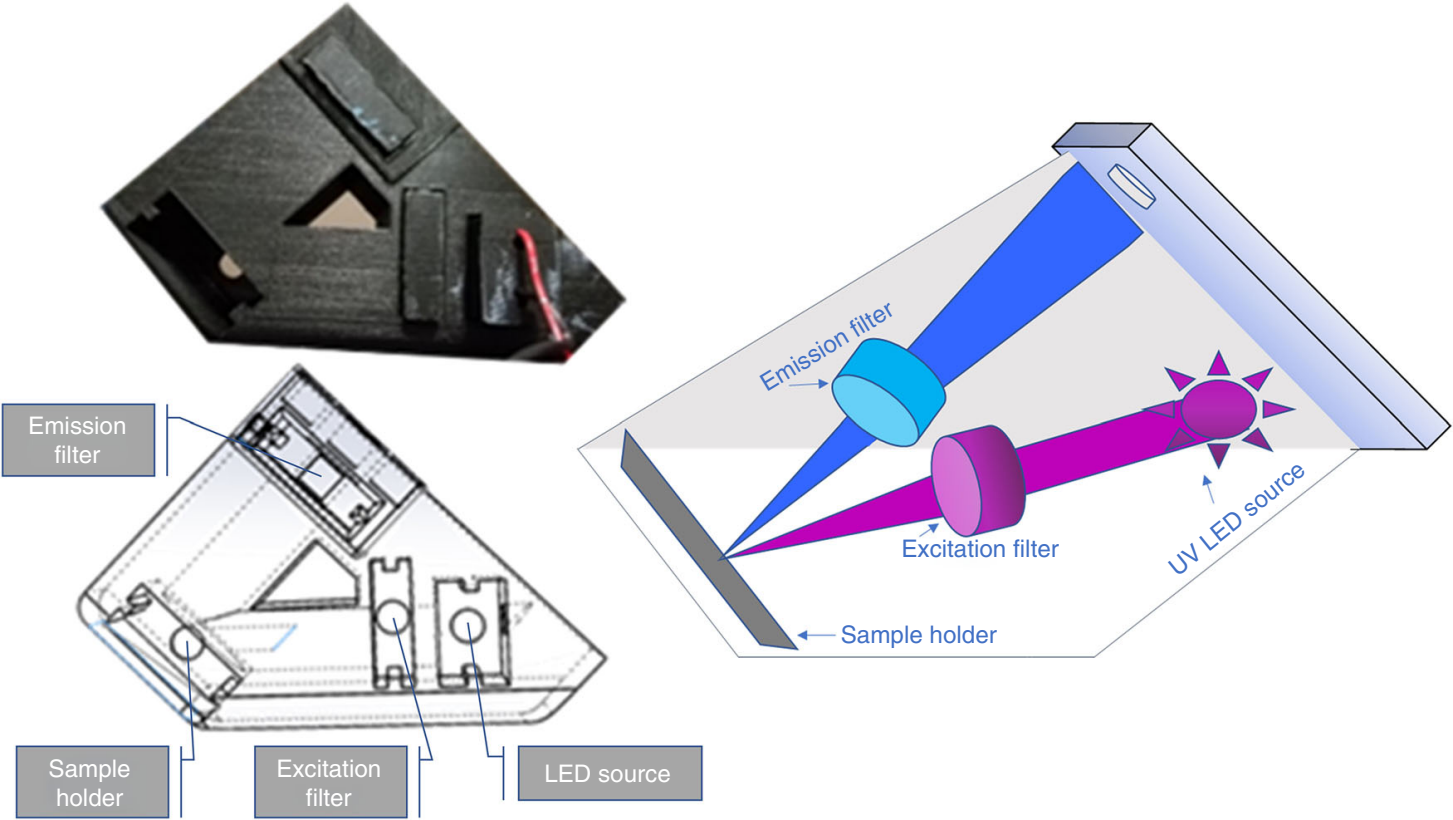
Detailed drawings of the reflection-mode fluorescent imaging device

A customized 3D-printed apparatus was developed which connected seamlessly with a predesigned smartphone case in order to take advantage of the sensitive smartphone camera for imaging. A 90° reflectance setup was utilized, incorporating a high-power LED, excitation filter, emission filter, and sample holder. A diagram of the finished 3D-printed handheld device is shown below in Fig. 6. This figure demonstrates the appearance, in both physical and wireframe form, alongside a sketch which indicates the light path and location of excitation and emission filters. The light source is composed of a UV LED, focused through a lens to a spot size equivalent to the field-of-view visible through the smartphone camera. An excitation filter and emission filter, each ½ inch diameter, are placed in the excitation and emission paths, respectively. Specific discussion of assembly is provided in the Supplementary.

The fluorescent sensor is measured through induction of fluorescent events, and hence requires a strong excitation light source at a particular wavelength, after which it will emit the fluorescence signal at a separate wavelength. For the purpose of data interpretation, it is critical to ensure that the collected photons are only emitted photons and not illumination photons. Since the excitation and emission light sources are at two distinct wavelengths, we can utilize emission and excitation optical filters sequentially to ensure the collected light is only the fluorescence, which is proportional to the concentration of (Pi) in the sample. The excitation filter guarantees that the excitation light is only provided at a narrow range of wavelengths, while the emission filter ensures that the illumination light is rejected while the excitation light is allowed to transmit. Hence, the combination of these two filters ensures that the detected light is only emission light. Calibration is carried out through the measurement of our standard solutions.

The 3D-printed device platform was designed using Solidworks and utilized a cassette system that held the LED, Filters and Paper Strip Holder. All parts were printed using a Makerbot-brand 3D printer. The cassettes could be readily removed, allowing insertion or replacement of the various components. As described previously, the entire device was designed to be extremely low cost to ensure the overall affordability of the system. Specifically, both filters had an OD (optical density) of 4 with the excitation filter being a 440-nm blocking edge BrightLine^®^ short-pass filter (Part Number: FB430-10, Thor Labs) and the emission filter a longpass filter, Cut-On Wavelength: 450 nm (Part Number: FEL0450, Thor Labs). Furthermore, the LED, a 5-Watt 430 nm Hyper-Violet LED was driven using a low-cost PowerPuck DC LED Driver (LEDSupply, Part# 02008B). This allowed for reliable current regulation from a stable 4.3 V DC source.

The sample pad was designed through the printing of a circular wax pattern onto Whatman™ 1 Chromatography Paper (Whatman™ 3001917, Fisher Scientific) using a Xerox Colorqube Wax Printer. The circular pattern was designed in a computer using MS Word 2016 and printed with the wax substrate to generate hydrophobic zones, entrapping the sensor within a defined volume. The paper was baked in an oven at 220 F for 3 min prior to applying the sensor in order to melt the wax through the paper, creating a continuous hydrophobic cylindrical barrier around the measurement zone. Once the barrier was formed, the fluorescent MDCC–PBP complex was immobilized through simple deposition and adsorption onto the strip, with the circular hydrophobic barrier effectively preconcentrating it into the sample zone.

Optimization of smartphone parameters required specific calibration of shutter speed and focal distance. Shutter speed of the smartphone reflects how long the shutter of the smartphone camera remains open and exposed to the fluorescent light. This is an important characteristic to control, since fluorescent emission is often very weak, but can be successfully collected if the shutter is kept open for a few seconds. However, it was also necessary to ensure that the shutter speed chosen provided sufficient signal to capture the lower concentrations of our linear curve while also preventing saturation of the signal at higher concentrations. Hence, this parameter must be carefully controlled to maximize our dynamic range. Focal distance controls the degree to which the focal plane of the smartphone is optimally aligned with the disposable wax-printed paper strip sample. This is a critical component in the experimental setup since the quantitative analysis requires a clear view of the fluorescent zone. If the focus is incorrect and the resulting image is blurred, the final analysis will suffer from a lower signal to noise ratio due to greater difficulty in segmentation of the sample zone from its border. To control these parameters precisely, the smartphone utilized the FV-5 Lite application, which allowed for control over ISO, focus and shutter speed. Specifically, ISO 800 and a 1/20 shutter speed were chosen in all experiments after sequential optimization steps. The “auto mode” of the typical smartphone camera is insufficient for our purposes since it is designed to be used in nonscientific applications. This mode includes undesired features, such as white balancing which alter the individual pixel values and reduce the integrity of the resulting data. For this reason, it is critical to collect the raw, unmodified data with careful control of shutter speed and focal distance—a set of parameters that the “auto mode” cannot adequately provide.

In addition to the previously described materials, reagents were purchased for interfering test assays. These include sodium chloride, fluoride, magnesium chloride, potassium chloride, and nitrates solutions, which were purchased from Fisher Scientific. The interfering agents were diluted as follows: NaCl (3 mM), NO_3_- (3 mM), MgCl_2_ (0.3 mM), F (3 mM), and KCl (0.3 M) and the volume of the interfering agent and (Pi) solution were kept the same during the interfering agent’s test. For this preliminary work, we utilized as a guide a similar paper utilizing fluorescent detection of phosphate to decide on our interfering agent’s concentrations^39^. The specific agents and their concentrations were chosen based on that work in order to better understand how our technique compared with interferences of similar fluorescent approaches. A detailed table of the reagents and their purity can be found in the Supplementary information.

## Supplementary information

Supplementary Information
