# Supplementary material for: Smart-phone, paper-based fluorescent sensor for ultra-low inorganic phosphate detection in environmental samples
Source: Microsyst Nanoeng. 2019 Oct 21;5:56. doi: 10.1038/s41378-019-0096-8 (PMC6803704; doi:10.1038/s41378-019-0096-8)
Supplement: Supplementary file 1 — Supplementary Information [file 41378_2019_96_MOESM1_ESM.pdf]

Table S-1. List of Reagents Utilized

Reagent	Purity	Purpose	Manufacturer	Catalog #
Phosphate Standard	TraceCERT®, Ion Chromatography Standard	Phosphate Source	Sigma Aldrich	38364
Deionized Water	ASTM Type 1	Dilutent	Fisher Scientific	LC267406
Nitrate IC Standard	Ion Chromatography Standard	Interfering Agents Test	Fisher Scientific	R5307900120
Magnesium Chloride	Molecular Biology Grade	Interfering Agents Test	Fisher Scientific	AM9530G
Potassium Chloride	Reagent Grade	Interfering Agents Test	Fisher Scientific	AA43398AK
Sodium Chloride	'Pure' Grade	Interfering Agents Test	Fisher Scientific	AC387640010
Fluoride	Ion Chromatography Standard	Interfering Agents Test	Fisher Scientific	AS-F9-1Y

Assembly & Measurement Details:

Once all of the necessary components have been prepared, the device is assembled for use, which is highlighted in Figure S-1 below. First, the smartphone is attached to a customized case, which utilizes three small plastic cylinders to securely fit into the device, aligning the camera with the light path of the device. Once assembled, the phone is aligned with the device and the imaging plane is in alignment with the location of the paper strip. The test sample is adsorbed within the sample pad of the wax-printed chromatographic paper strip and is then slid

into the light-path through a removable cassette. Since the liquid sample is deposited into the sample zone through a single drop from a pipette, under UV illumination the fluorescent signal appears as a distinct circle, which is automatically extracted from the image through a custom Matlab script. Collected images were analyzed in ImageJ in order to separate the individual RGB channels and calculate fluorescent density. Only the blue channel was analyzed as it is most responsive to the blue fluorescence of the sensor, with pixel values corresponding to fluorescence intensity. Since we are collecting images using an RGB camera, we utilize an open-source platform called ImageJ to separate the individual red, green and blue channels of the color image. The fluorescence of our sensor, being blue, only provides an image in the blue channel, which is extracted for later analysis. In order to provide standardized assessment, the Hough Circular Transform was used within Matlab through the *imfindcircles* function, which automated the identification of the circular fluorescent object and the calculation of the average interior pixel value.

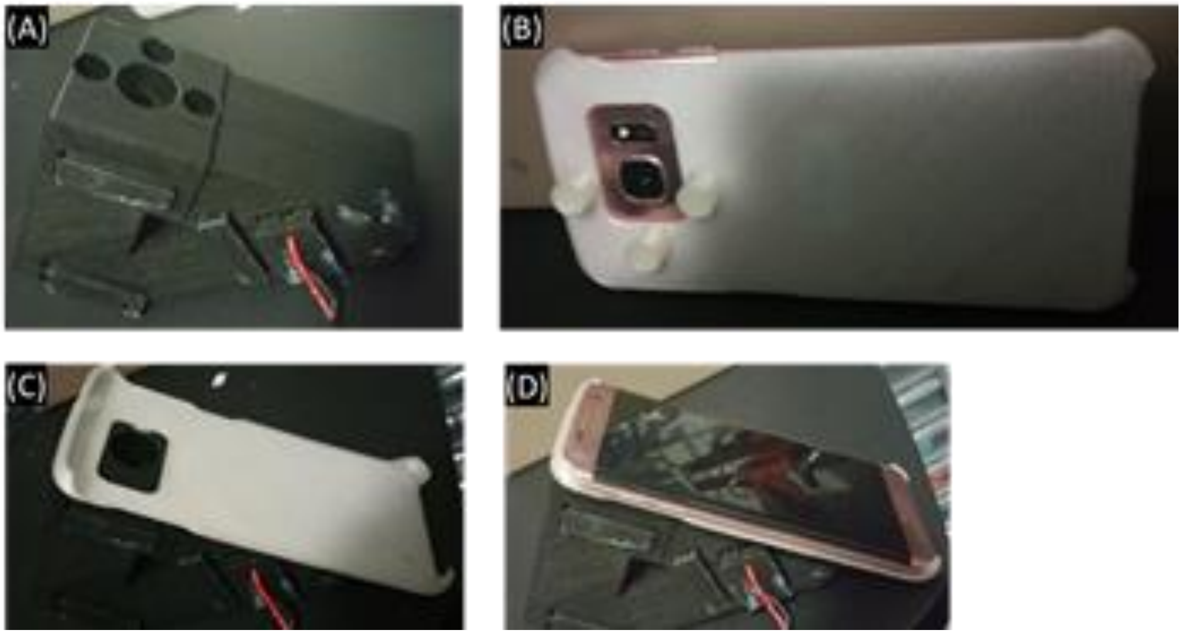

Figure S-1: Assembly of fluorescence imaging system. (A) Fluorescence Measurement Chamber (B) Smart-phone Adapter (C) Connection of Chamber to Adapter (D) Complete Assembly

Paper-Strip Fabrication

A wax printer is a specialized printer that heats and deposits wax onto a paper substrate, similarly to how inkjet printers use ink. However, wax printers are extremely useful since the wax they print is hydrophobic, allowing for the creation of paper-fluidic reservoirs when a ring is printed. Furthermore, the wax is printed onto porous chromatographic paper, and after printing the paper is baked to melt the wax thoroughly through the paper, generating a solid hydrophobic barrier around an isolated cylindrical sample zone. This is highlighted in the figure below. This sample zone is first introduced to the fluorescent sensor, which is immobilized via passive adsorption to the surface. Wetting with the aqueous sample can be then carried out and the result measured. Storage of the functionalized paper strip is recommended at refrigerated temperatures to maximize fluorophore stability. The original paper strip was designed to have a 0.3-inch diameter circle, with 3 pt. thickness. The strip was melted in an oven at 220oF for 3 minutes. Following baking, the inner diameter fell to 0.2 inch.

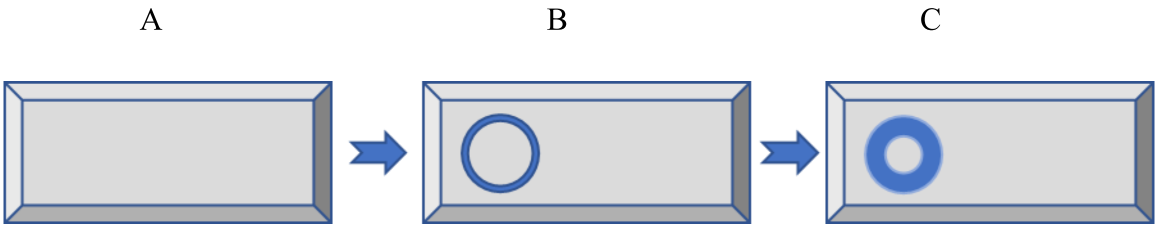

Figure S-2: Formation of the paper strip platform. (A) Chromatographic paper is chosen due to its porosity and capability for passive protein adsorption. (B) The wax printer begins by printing a simple wax ring on top of the chromatographic paper. (C) After heating the chromatographic paper through use of an oven, the ink melts axially through the paper, creating a continuous hydrophobic cylindrical barrier throughout the thickness of the paper strip.
